# Synaptic boutons sizes are tuned to best fit their physiological performances

**DOI:** 10.1186/1471-2202-14-S1-P138

**Published:** 2013-07-08

**Authors:** Markus M Knodel, Dan Bucher, Romina Geiger, Lihao Ge, Alfio Grillo, Gabriel Wittum, Christoph Schuster, Gillian Queisser

**Affiliations:** 1 Goethe Center for Scientific Computing, Frankfurt University, Germany; 2 Bernstein Group for Computational Neuroscience, Heidelberg University, Germany; 3 Interdisciplinary Institute for Neuroscience, Heidelberg University, Germany; 4 EMBL Heidelberg, Germany; 5 Dept. of Mathematical Sciences, Polythecnic of Turin, Italy

## 

To truly appreciate the myriad of events which relate synaptic function and vesicle dynamics, simulations should be done in a spatially realistic environment. This holds true in particular in order to explain as well the rather astonishing motor patterns which we observed within in vivo recordings which underlie peristaltic contractionsas well as the shape of the EPSPs at different forms of long-term stimulation, presented both here, at a well characterized synapse, the neuromuscular junction (NMJ) of the Drosophila larva (c.f. Figure 1). To this end, we have employed a reductionist approach and generated three dimensional models of single presynaptic boutons at the Drosophila larval NMJ. Vesicle dynamics are described by diffusion-like partial differential equations which are solved numerically on unstructured grids using the uG platform. In our model we varied parameters such as bouton-size, vesicle output probability (Po), stimulation frequency and number of synapses, to observe how altering these parameters effected bouton function. Hence we demonstrate that the morphologic and physiologic specialization maybe a convergent evolutionary adaptation to regulate the trade off between sustained, low output, and short term, high output, synaptic signals. There seems to be a biologically meaningful explanation for the co-existence of the two different bouton types as previously observed at the NMJ (characterized especially by the relation between size and Po), the assigning of two different tasks with respect to short- and long-time behaviour could allow for an optimized interplay of different synapse types. We can present astonishing similar results of experimental and simulation data which could be gained in particular without any data fitting, however based only on biophysical values which could be taken from different experimental results. As a side product, we demonstrate how advanced methods from numerical mathematics could help in future to resolve also other difficult experimental neurobiological issues.

**Figure 1:**
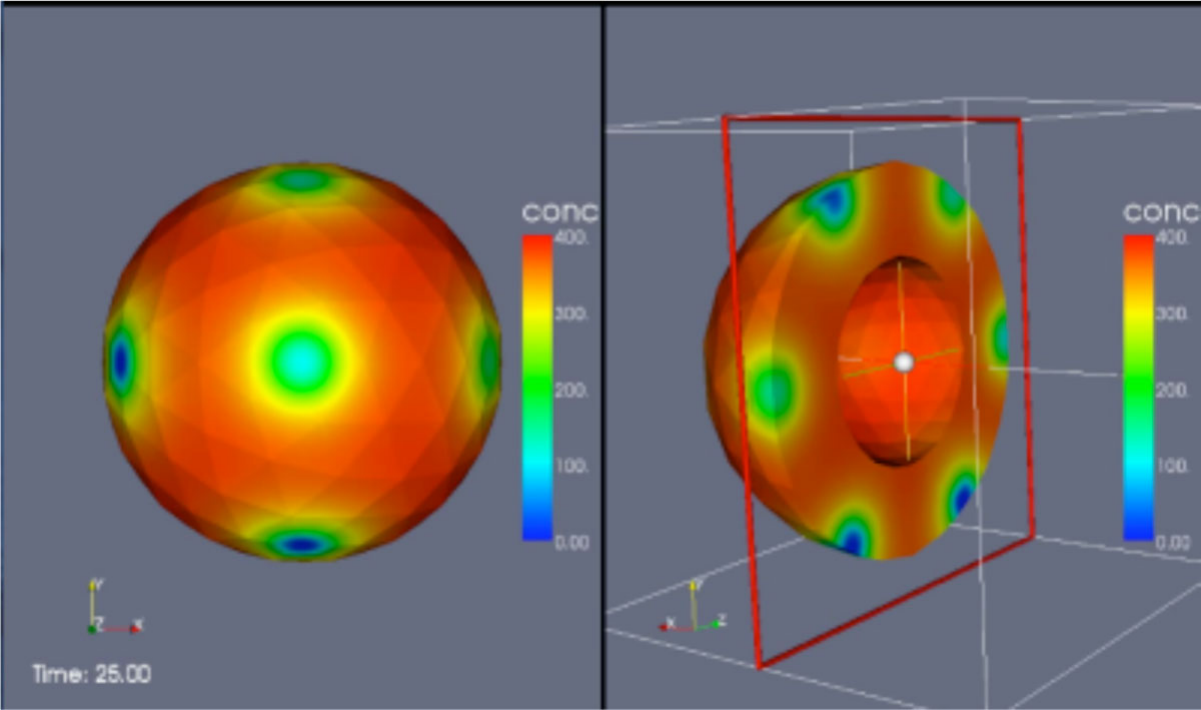
Simulation of a bouton of the Drosophila NMJ

## References

[B1] Jan L Jan Y Properties of the larval neuromuscular junction in Drosophila melanogaster J Physiol 1976 262 1 189 214 11339 10.1113/jphysiol.1976.sp011592 PMC1307637

[B2] Schuster C Davis G Fetter R Goodman C Genetic dissection of structural and functional components of synaptic plasticity. ii fasciclin ii controls presynaptic structural plasticity Neuron 1996 17 4 655 67 doi: 10.1016/S0896-6273(00)80198-1 10.1016/S0896-6273(00)80198-1 8893023

[B3] Delgado R Maureira C Oliva C Kidokoro Y Labarca P Size of vesicle pools, rates of mobilization, and recycling at neuromuscular synapses of a Drosophila mutant, shibire Neuron 2000 28 941 53 doi: 10.1016/S0896-6273(00)00165-3 10.1016/S0896-6273(00)00165-3 11163278

[B4] Bastian P Birken K Johannsen K Lang S Reichenberger V Wieners C Wittum G Wrobel CA W. Jäger and E. Krause High performance computing in science and engineering 1999 Springer 326 339 Parallel software-platform for solving problems of partial differential equations using unstructured gr ids and adaptive multigrid methods

